# A method to identify respiratory virus infections in clinical samples using next-generation sequencing

**DOI:** 10.1038/s41598-018-37483-w

**Published:** 2019-02-22

**Authors:** Talia Kustin, Guy Ling, Sivan Sharabi, Daniela Ram, Nehemya Friedman, Neta Zuckerman, Efrat Dahan Bucris, Aharona Glatman-Freedman, Adi Stern, Michal Mandelboim

**Affiliations:** 1 School of Molecular Cell Biology and Biotechnology, George S. Wise Faculty of Life Sciences Tel Aviv University, Tel Aviv, Israel; 2 Central Virology Laboratory, Ministry of Health, Chaim Sheba Medical Center, Tel-Hashomer, Ramat-Gan, Israel; 3 Department of Epidemiology and Preventive Medicine, School of Public Health, Sackler Faculty of Medicine, Tel-Aviv University, Tel-Aviv, Israel; 4 The Israel Center for Disease Control, Israel Ministry of Health, Tel-Hashomer, Israel

## Abstract

Respiratory virus infections are very common. Such infections impose an enormous economic burden and occasionally lead to death. Furthermore, every few decades, respiratory virus pandemics emerge, putting the entire world population at risk. Thus, there is an urgent need to quickly and precisely identify the infecting agent in a clinical setting. However, in many patients with influenza-like symptoms (ILS) the identity of the underlying pathogen remains unknown. In addition, it takes time and effort to individually identify the virus responsible for the ILS. Here, we present a new next-generation sequencing (NGS)-based method that enables rapid and robust identification of pathogens in a pool of clinical samples without the need for specific primers. The method is aimed at rapidly uncovering a potentially common pathogen affecting many samples with an unidentified source of disease.

## Introduction

Respiratory virus infections are very common around the world, especially during the winter season. Such infections impose an enormous economic burden and can lead to death, especially among the elderly. Respiratory virus infections are very easily transmitted from one person to another and cause a global pandemic every few decades^1,2^. Thus, to prevent their rapid spread and minimize mortality rates, it is essential to diagnose infection as soon as possible.

Several laboratory methods, such as enzyme-linked immunosorbent assay, nucleic acid hybridization, and polymerase chain reaction (PCR), are utilized to detect respiratory virus infections^3^. However, these assays are highly dependent on previous knowledge of the virus, and thus are ineffective in identifying new pathogens and are less effective in identifying novel variants of a known pathogen^4^. Mainly, these methods rely on knowledge about the viral sequence at hand, and hence relevant nucleic acid probes (primers) are used. Accordingly, rare viruses that are not commonly examined in the laboratory, will not be detected using these conventional methods.

Next-generation sequencing (NGS) technologies enable simultaneous sequencing of large numbers of samples, and can be used for the unbiased detection and characterization of multiple agents in a single sample. Use of NGS eliminates the need for prior knowledge of virus genomic sequences and provides advantages over traditional methods such as PCR amplification or microarray hybridization, that are dependent on target-specific primers^3^. To date, NGS has been instrumental in the discovery of novel viruses and characterization of viral communities. NGS was used to discover a new arenavirus^5^, a new ebola virus^6^ and zika virus^7^. NGS is also applied for the characterization of viruses in the environment^8,9^, in animals^10^ and in humans^11,12^. Here, we describe a new NGS-based method designed to identify viruses in a pool of clinical samples.

## Materials and Methods

### Patients and samples

As part of community influenza surveillance, conducted in collaboration with the Israel Center for Disease Control (ICDC), clinical combined nose throat swab samples were collected from 300 patients presenting with influenza-like illness (ILI), during the winter season spanning between October 2013 and December 2013. The samples were collected using ∑-Virocult® M40-A Compliant kit (MWE, UK), suspended in 2 ml M40 medium, and stored up to 48 hours in 4 °C. Following preparation, samples were stored in −70 °C. 500 microliters were used in the first preparation and the rest was kept in −70 °C. Then, for step 3 (Fig. 2), we used the original 54 samples and controls that were stored in −70 °C.

### Ethical considerations

Community influenza surveillance, including combined nose throat samples, is performed under the Public Health Ordinance enacted in Israel. The IRB of the Sheba Medical Center approved the research: Helsinki Number 4379-17-SMC. Informed consent is not required.

### Nucleic acid extraction and qRT PCR

Total nucleic acid content was extracted from 500 microliters of the Virocult medium using NucliSENS easyMAG (BioMerieux, France). The presence of respiratory viruses was determined by TaqMan Chemistry using the ABI 7500 instrument. As part of community influenza surveillance, RNA viruses detection was performed using a panel of real-time reverse transcription-polymerase chain reaction (rRT-PCR) assays, as previously described: influenza A and B^13^, influenza A(H1N1)pdm09^14,15^ and respiratory syncytial virus (RSV)^16^. Then, samples were tested for presence of: human metapneumovirus^17^, enterovirus^18^, rhinovirus^19^, parainfluenza 3^20^ and coronaviruses^21^. RT-PCR was performed to detect DNA virus adenovirus^22^ and bocavirus^23^. For the RNA rRT-PCR assays, the Ambion Ag-Path master mix (Life Technologies, Carlsbad, CA) was used, whereas for the DNA assays, ABgene Absolute Blue (Thermo, UK) was used. Both were used according to the manufacturer’s instructions.

Torque teno virus (TTV) was detected by qRT-PCR^24^, and parainfluenza 1 virus was detected by RT-PCR, as previously described^25^. Cucumber green mottle mosaic virus (CGMMV) was detected using the following primers: forward 5′ATGGCAAACATTAATGAACAAATCAA3′ and reverse 5′TCTATCTGGAAAGTTGGAAGAGGTC3′

The bacteria *Streptococcus pneumoniae* was detected by PCR as previously described^26^ and using the following primers: forward 5′AAATGCGCGGTGAAGCAAAAGG3′ and reverse 5′GACCAGTAGCAGCTTGGAAACG3′. *Staphylococcus aureus* was detected by PCR as previously described^27^ and using the following primers: forward 5′CAAAAGACTGGGGTAAACGTCGC3′ and reverse 5′CGGTCCGTTTGCATTTGCAAATGG3′. *Pseudomonas putida* was detected by PCR as previously described^28^ and using the following primers: forward 5′GACAGTCGCTGCCTGTTTTTG3′ and reverse 5′AGTCGATCTGTAAGCCGGGTTTTG3′. Influenza C virus^29^ and Parainfluenza 4 virus^30^ were detected by RT-PCR on the samples after the second run of RNA library.

### Sample preparation

200 microliters of the original samples that were stored in −70 °C in the Virocult medium were freeze-thawed three times and then centrifuged at high speed for 20 minutes. OmniCleave Endonuclease (250U; Epicenter Distributor Madison, WI USA, OC7850K) was added to the supernatants in the presence of 2.5 mM MgCl_2_ for 1 h at 37 °C. The genome was extracted from the samples using NucliSENS easyMAG. RNaseP content, quantified by qRT-PCR, served as an indicator of the amount of human genome remaining in the sample^31^.  All samples in which the RNaseP was above 35 Ct qRT-PCR were pooled together.

### Library preparation and sequencing

The Agilent RNA 6000 Nano Kit (Life Technologies, Waldbronn, Germany) was used to evaluate total RNA quality. RNA concentration was measured using the Qubit RNA BR Assay kit (Life Technologies). The library was processed for NGS sample preparation using SMARTer Stranded RNA-Seq Kits (Clontech, a Takara Bio Company, CA, USA), Strand-Specific Library Construction for Transcriptome Analysis on Illumina Platforms.

The Agilent High Sensitivity DNA Kit with the Agilent 2100 Bioanalyzer (Life Technologies, Waldbronn, Germany) was used to size, quantify and quality control DNA sequencing libraries. NGS was performed using a MiSeq v2 kit (500 cycles) (Illumina, San Diego, CA). After automated cluster generation in MiSeq, the sequencing was processed and genomic sequence reads were obtained.

For the detection of RNA viruses, sample extracts were pretreated with RNase free DNase (promega), the Agilent RNA 6000 Nano Kit (Life Technologies, Waldbronn, Germany) was used to evaluate total RNA quality. RNA concentration was measured using the Qubit RNA HS Assay kit (Life Technologies). RNA libraries were constructed for NGS using SMARTer Stranded RNA-Seq Kit according to manufacturer instructions (Clontech, a Takara Bio Company, CA, USA).

For DNA viruses, sample extracts were pretreated with RNase (JGI), DNA concentration was measured using the Qubit DNA HS Assay kit. DNA libraries were constructed using Nextera XT kit (Illumina, San Diego, CA) according to manufacturer instructions. RNA and DNA libraries size distribution were analyzed using Tape Station 2.0 (Agillent). Libraries were normalized according to the median fragment size measured by Tape Station and library concentration measured by Qubit. Sequencing was processed with Miseq v3 kit (600 cycles) (Illumina, San Diego, CA). After automated cluster generation, the genomic sequence reads (FASTQ) files were obtained for further analysis.

### Computational analysis of NGS data

Bowtie 2.0^32^ was used to filter out reads that mapped either to: (a) the human genome (hg19) or to the human transcriptome, both downloaded from Ensembl (www.ensembl.org), or (b) to bacterial genomes listed in the human microbiome project (downloaded from https://hmpdacc.org/). Remaining reads were queried using Bowtie 2.0 against a virus-only database (downloaded from NCBI - ftp://ftp.ncbi.nih.gov/refseq/release/viral/) and the hits were validated with blastn and blastx. We omitted reads that mapped to viruses with bowtie2 but did not map to the same virus with blast. In order to examine reads that did not map to any database described above, we used the Velvet de-novo assembly program^33^ with default settings to create larger “contigs” of data. Finally, contigs were queried against the nt (non-redundant nucleotide) and nr (non-redundant protein) databases at NCBI, using blastn and blastx, respectively, with default parameters.

## Results

### Sample selection and work outline

**Figure 1:**
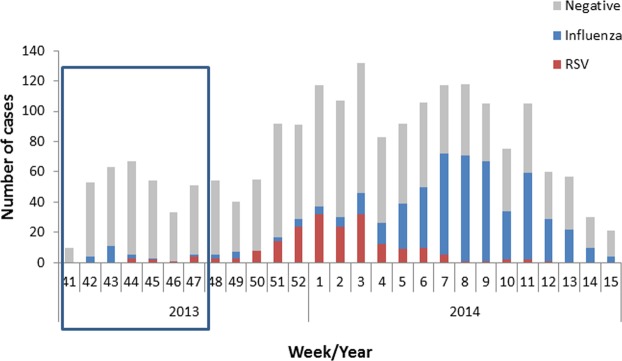
RSV and influenza infection throughout the winter season. The number of clinical samples infected with influenza virus or RSV in the 2013–2014 winter season in Israel. The week number and year are shown on the X axis. The 300 samples addressed in this paper are highlighted in the box.

In the beginning of the winter season, the identity of the viruses causing ILS, other than RSV and influenza, was unknown (Fig. 1). Later in the winter two epidemic waves were noticed, RSV and then influenza. However, a higher percentage of patients still suffered from a respiratory infection of unknown etiology, with clinical symptoms compatible with viral infection (Fig. 1). We therefore set out to develop a general and robust approach to identify the unknown viruses responsible for ILS. Three possibilities were considered: (1) The cause of the ILS is an unknown virus or a known virus not yet associated with respiratory illnesses; (2) the cause of the ILS is a virus not commonly tested in the laboratory; (3) the cause of the ILS is a known virus that causes respiratory infection that cannot be identified in the routine RT PCR tests due to variability.

**Figure 2:**
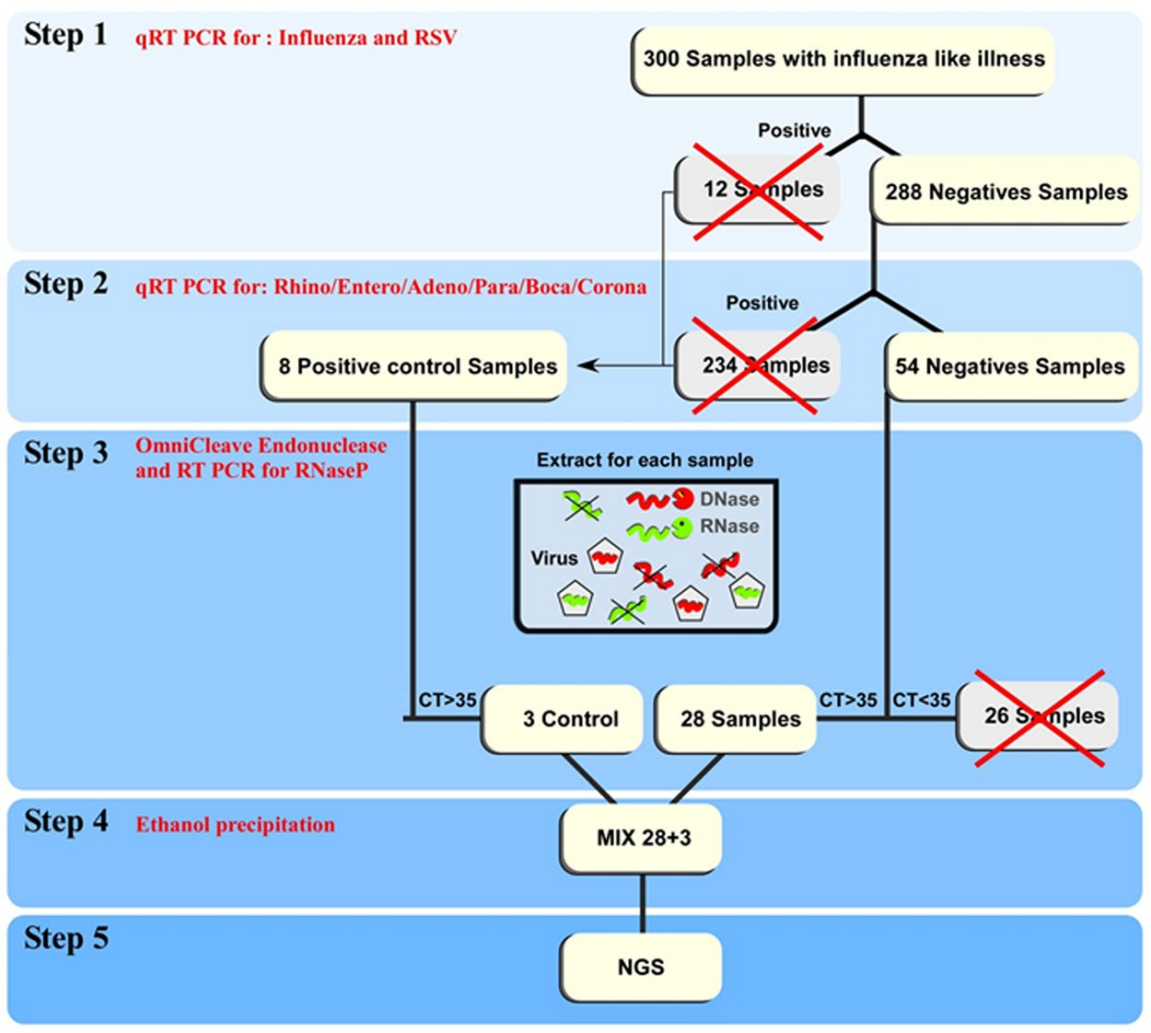
Schematic presentation of the workflow of the described method.

**Figure 3:**
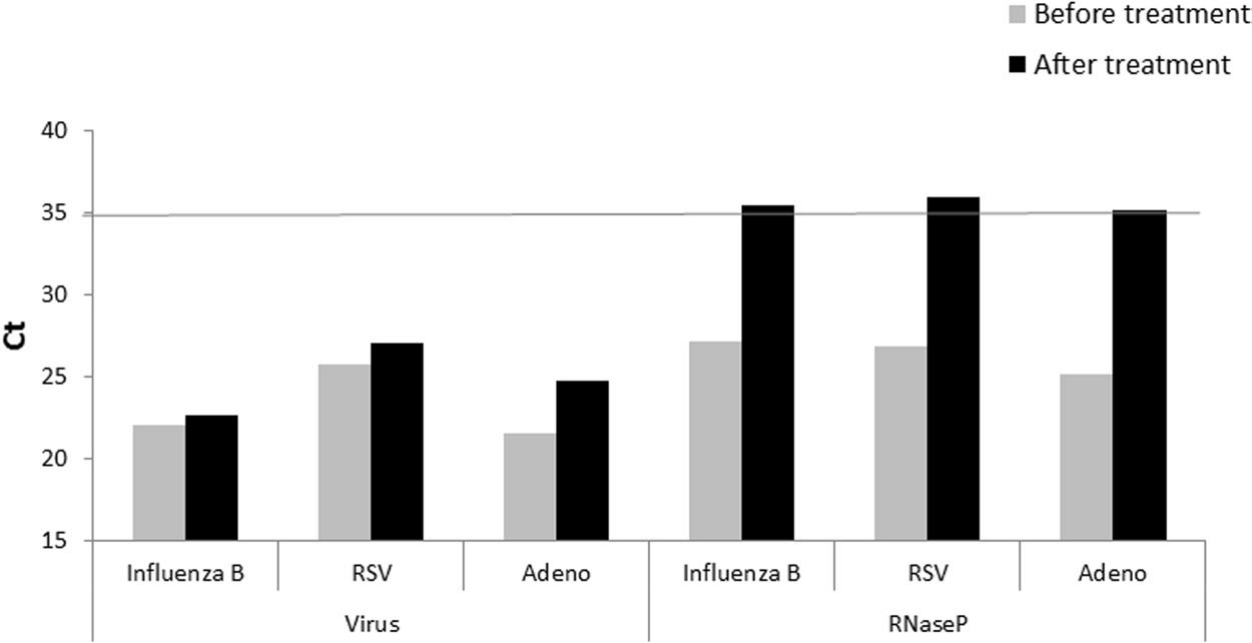
qRT-PCR for viruses and RNaseP. Ct results before and after Omnicleave treatment for the 3 controls tested viruses and for RNaseP.

The workflow of the approach is shown in Fig. 2. Initially, qRT-PCR for influenza and RSV was performed on 300 samples obtained from the beginning of the 2013–2014 winter season (Fig. 1); 12 samples were positive. The remaining 288 samples (Fig. 2), were then assayed by qRT-PCR for: rhinovirus, enterovirus, adenovirus, parainfluenza 3, bocavirus and coronaviruses; 234 samples were found to be positive (Fig. 2). In the next step, the remaining 54 samples and 8 positive control samples (2 influenza A, 2 influenza B, 2 RSV and 2 adenovirus) were treated with OmniCleave, which destroys free DNA and RNA, leaving the viral genome unaffected, as it is protected by a capsid. qRT-PCR of all samples for the cellular gene RNaseP was performed. Following treatment, the control virus amount remained unchanged, while RNaseP levels were reduced (resulting in higher cycle threshold (Ct) Fig. 3). The treatment efficiency threshold was set at Ct RNaseP > 35. We selected Ct > 35 since this indicates low levels of human genome that will not interfere with the viral identification. A level of Ct > 35 is equivalent to less than one copy of human DNA. The Ct of all control viruses, but two, was above 35. In one influenza B sample the Ct was 34.01 and in one adenovirus sample the Ct was 34.81. For the mixed sequencing we took one sample of each control virus.

Following this, 28 clinical samples were left in addition to 3 control samples (Influenza B, RSV and adenovirus) (Fig. 2). The samples were pooled and NGS was performed (Fig. 2).

### Next-generation sequencing of the pooled samples

The resulting NGS data consisted of ~9 M paired-end reads of 150 bp DNA fragments. Reads similar to human or known bacterial genomes were filtered out, and the remaining reads were tested for similarity to viruses from known databases, as previously done^11,34^. Among the 9M reads, 27% found to be human genomic material, and ~61% mapped to bacterial genomes were filtered out, leaving ~1.1 million reads to be further analyzed. When applying Bowtie 2.0 to match the remaining reads to known viruses, approximately 34,000 mapped to known viruses, with 29,000 reads mapping to phi X 174 bacteriophage, which is a control sample of Illumina sequencing. The remaining ~1 million unmapped reads were assembled into larger segments (contigs). Assuming that reads from a pathogen will be abundant enough in the sample to create somewhat long contigs, 8387 contigs with more than 350 nucleotides were detected and used to query the non-redundant nucleotide and protein databases in NCBI. The vast majority of contigs mapped to a bacterial or a phage source. The remaining viral-mapped contigs were mapped to influenza B and Torque teno virus (TTV) and one contig mapped to human picobirnavirus. There was a small number of contigs that mapped to plant, fungi, animal and protists.

### Detection of known viruses

**Figure 4:**
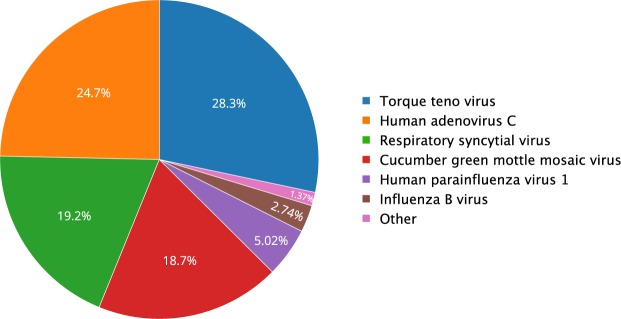
Viruses identified by NGS in 2013–2014 pooled clinical ILS samples. The chart includes viruses with more than 30 reads mapped to them, and read counts per virus are normalized by genome length of the virus.

We next focused on the 5,000 reads that mapped to known viruses. Of these, ~2000 mapped to bacterial phages and ~3000 to other viruses. From the latter reads, three viruses (adenovirus, RSV and influenza B) that were included as positive samples in our pooled sample, were detected (Fig. 4). Notably, the average coverage of the positive control viruses was quite low: sixteen reads per base for adenovirus and one read per base for RSV and influenza B. Additional viruses identified included parainfluenza virus 1, TTV, a virus which is frequently present in humans, and a cucumber green mottle mosaic virus (CGMMV), present in vegetables like cucumber and watermelon and is not known to cause illness in humans (Fig. 4 and Table 1).

**Table 1:** Number of reads for each virus.

Virus	Number of mapped reads
Human adenovirus C	1904
Respiratory syncytial virus	627
Cucumber green mottle mosaic virus	258
Human parainfluenza virus 1	161
Torque teno virus	122
Torque teno mini virus	78
Influenza B virus	78
Torque teno midi virus	12
Moloney murine leukemia virus	6
Apis mellifera filamentous virus	5
Human herpesvirus 7	5
Emiliania huxleyi virus 86	4
Megavirus chiliensis	3
Prunus necrotic ringspot virus	2
Tomato brown rugose fruit virus	2
Tobacco mosaic virus	2
Human herpesvirus 4	2
Bovine respiratory syncytial virus	2
Avian leukosis virus - RSA	1
Chrysodeixis chalcites nucleopolyhedrovirus	1
Fowlpox virus	1
Human papillomavirus type 5	1
Human papillomavirus type 9	1
Variola virus	1

Number of mapped reads for each virus discovered in the next generation sequencing.

**Figure 5:**
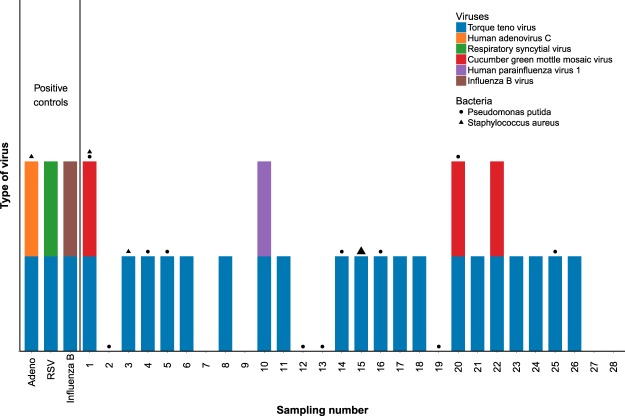
Verification of virus and bacteria presence in each of the samples. The presence of each of the viruses identified (Fig. 4) was verified in each sample by qRT-PCR/PCR. Viruses are shown in the colored bars and bacteria are shown with the shaped symbols, the size of the symbol corresponds with the strength of detection.

To validate that the newly identified viruses were indeed present in the tested samples and to determine precisely which sample was infected with each of these viruses, qRT-PCR was then performed on the individual samples. As can be seen in Fig. 5, TTV was detected in 23 of 31 samples (20 unidentified samples and the 3 control samples). TTV titer was high in two unidentified samples and low in the rest of the samples detected.

The control viruses that were included in the pooled assay sample were identified only in the control samples (Fig. 5). One patient (number 10, Fig. 5) was infected with parainfluenza 1 virus and three patients with CGMMV, that is not known to cause illness in humans (Fig. 5). Human picobirnavirus was detected in one of the contigs assembled. This virus causes gastroenteritis symptoms and is not associated with ILS^35^.

### Detection of possible bacterial agents

Although our study is focused on patients with viral symptoms rather than bacterial symptoms it is reasonable to assume that some of the patients were infected with bacterial agents. Our sequencing resulted in ~5.4 million reads that were mapped to bacteria. 50% of the bacterial reads were mapped to the *Pseudomonas* genus, which is mostly non-pathogenic and if pathogenic it is usually due to opportunistic infection^36^. The other genus detected with high prevalence was *Streptococcus* with 22% of the reads. Some species from this genus such as *Streptococcus pneumoniae* (216 K reads) can cause disease with influenza like symptoms. Another possible causative agent of ILS that was detected is *Staphylococcus aureus* that had 170 K reads mapped to it. It is important to acknowledge that the bacterial results obtained here do not represent the full repertoire of bacteria in the samples due to the OmniCleave treatment that destroyed most of the bacterial genomes.

To determine which samples were infected with each of these bacterial strains we performed PCR on the individual samples. *Pseudomonas putida*, which was the most prevalent Pseudomonas species found, was present in 12 samples in low quantities; *Staphylococcus aureus* was detected with a strong signal in one sample (patient 15, Fig. 5) and with a weak signal in two other samples and in the adenovirus positive control. *Streptococcus pneumoniae* was not detected in our samples (Fig. 5). These results imply that at least in one patient (sample 15) the ILS symptoms may have been a result of bacterial infection by *Staphylococcus aureus*. It is quite possible that there are more bacterial strains that cause ILS symptoms in our samples that were not detected.

### Improvement of the method

We were concerned that our initial >35 cutoff for sample inclusion might have been too stringent so we performed a second round of NGS sequencing, this time including all 54 negative samples. We split the pooled samples in two and created separate samples for RNA viruses and DNA viruses (using treatments of DNase and RNase respectively). We did this to maximize library quality and the probability for viral detection. The sequencing results for the RNA viruses sample revealed three additional viruses that were not detected in the first sequencing. Those viruses are parainfluenza 4 virus and influenza C virus that cause ILS symptoms and rotavirus A which causes diarrhoeal disease. Parainfluenza 4 and influenza C were identified by RT-PCR in two clinical samples (parainfluenza 4 in patient number 49 and influenza C in patient 30). The DNA viruses sample didn’t reveal presence of previously undetected viruses.

## Discussion

Infections with respiratory viruses are common and affect practically the entire population. Such infections impose a heavy economic burden, due to loss of working days, and they can be life-threatening. Thus, it is critical to monitor such infections from an early stage. However, occasionally the virus responsible for the infection cannot be identified. For example, we show here, that at the start of our screen, no common respiratory virus could be identified in 54 out of 300 (18%) samples collected from patients with ILS. Furthermore, new methods are needed to rapidly detect viruses present in a pool of samples to enable rapid treatment of the population in case of pandemics. Here, we presented a new NGS-based method to identify the viruses present in these samples.

In this method, all unknown samples were pooled with positive controls, enabling virus identification in a single experiment, rendering the approach rapid, robust, efficient and economic. It takes less than one day to perform the qRT-PCR and one day to generate the library for sequencing. The bioinformatics analysis runs for a few hours. Next, the presence of the identified virus in each of the samples can be rapidly determined using qRT-PCR.

Out of 54 samples that were sequenced, our method discovered infection with human parainfluenza 1 virus, human parainfluenza 4 virus and influenza C virus, each in one of the patients. The original clinical diagnosis of our samples was infection with a viral agent, based on their symptoms. Nevertheless, our method also detected a sample that was most likely infected with a bacterial agent rather than a viral one. Interestingly, TTV was identified in many samples. TTV is the first human virus identified to have a circular negative-stranded DNA genome^37^. Its genome is approximately 3.8k nucleotides (nt) long^38^. TTV frequently circulates in many geographic regions^24,38,39^. Although this virus has a very high prevalence in the general population, neither its interaction with its hosts nor its direct involvement in the etiology of specific diseases are fully understood. Nevertheless, a significant correlation between TTV loads and airflow limitation within the peripheral airways, as well as between severity of bronchiectasis and decrease of lung function, has been observed^40^. Thus, it is possible that TTV was the cause of the ILS. Further studies will be necessary to confirm that TTV is indeed responsible for the ILS and to understand why TTV is present in some individuals (even in the healthy) but not in others.

Detection of CGMMV demonstrates that the method can identify viruses present in human diet providing further indication of the validity and accuracy of the presented method.

The virus responsible for the infection could not be certainly identified in 51 samples. The following hypotheses may be raised: (a) the source of disease was bacterial rather than viral, as suggested herein for one sample that may have been infected with *Staphylococcus aureus*. If this is indeed the case then for the other unidentified samples, the bacterial DNA may have been destroyed when using the OmniCleave endonuclease (see Methods), (b) the source of the disease was TTV, (c) the source of the disease was viral but due to low virus titer it was undetectable. Accordingly, we suggest in the future improving the sample preparation process and striving for higher sequencing depth when aiming to discover a new virus or a novel virus variant.

In summary, we present a rapid and efficient method for detection of novel, uncommon and non-tested pathogens associated with disease that might be useful in a clinical setting. With the advancement of NGS technology, we expect this approach to become increasingly useful.
